# Addition of Capsicum oleoresin, Carvacrol, Cinnamaldehyde and their mixtures to the broiler diet II: Effects on meat quality

**DOI:** 10.1186/s40781-018-0165-9

**Published:** 2018-04-30

**Authors:** Hasan Hüseyin İpçak, Ahmet Alçiçek

**Affiliations:** Department of Animal Science, Faculty of Agricultural, Ege University, Bornova, 35100 Izmir, Turkey

**Keywords:** Broiler, Capsicum oleoresin, Carvacrol, Cinnamaldehyde, Meat quality

## Abstract

**Background:**

In recent years, with the prohibition of antibiotics used as growth stimulants in the nutrition of farm animals, researchers have searched for alternative natural and reliable products in order to be able to sustain the developments experienced during the use of antibiotics and to overcome the possible inconveniences. In this context, studies on evaluation of essential oils in poultry nutrition have been reported to improve the utilization of feed, stimulate the release of digestive enzymes, increase absorption in the stomach and intestines, antimicrobial and anti-parasitic effects and thus, can be an alternative to antibiotics and improve meat quality as well. Indeed, this study has been carried out to explore the effects of the addition of 150 mg/kg capsicum oleoresin (CAP), carvacrol (CAR), cinnamaldehyde (CIN) or their mixture (CAP+CAR+CIN) into the broilers’ ration over sensory, physical and chemical properties in breast meat and leg meat.

**Methods:**

Experiments were conducted over 400 male and female broiler chicks (Ross-308) in 5 groups (1 control group and 4 treatment groups), each composed of 80 chicks. The control group was fed without feed additives while the second, third, fourth and the fifth groups were fed with 150 mg CAP/kg feed, 150 mg CAR/kg feed, 150 mg CIN/kg feed, and 150 mg CAP+CAR+CIN/kg feed, respectively.

**Results:**

Addition of CAP, CAR, CIN or CAP+CAR+CIN had effects on the sensory (of taste, tenderness, juiciness and overall acceptability); physical properties (of L* value and toughness), the chemical properties (of DM, CF, CP, linoleic, EPA, behenic, MUFA, PUFA and ∑n-6 of the leg meat), the physical characteristics (of toughness and firmness), and the chemical properties (of CF, CP, linoleic, ecosenic, EPA, lignoseric, MUFA and ∑n-3) of the breast meat in comparison to control group. Furthermore, while the treatments had positive impacts on thawing loss, cooking loss and water holding capacity in both breast and leg meat; no effect was observed on pH value and lipid oxidation on day 1, day 4 and day 8.

**Conclusion:**

The results strongly suggested that the addition of CAP, CAR, CIN or CAP+CAR+CIN to the rations of the broiler chicks changed the sensory, physical and chemical properties of breast and leg meat. It was also observed that these compounds were more effective when they were added to the ratio as a mixture rather than adding them individually.

## Background

In recent years, foremost issues that have been interesting to all the world countries are food safety, food security and nutrition [1]. The essential requirement, probably the most important one, of individuals is nutrition to live healthy and strong in terms of physically, mentally and emotionally, to improve themselves economically and socially, for prospering and to continue themselves existence as happy, peaceful and secure [2]. It is asserted that an adult person should take 1 g of protein per kg of body weight per day in order to keep healthy and balanced diet and 42–50% of this amount should consist of animal originated proteins [3]. Chicken meat is one of the most important resources of animal protein that should be consumed for physical and mental development, healthy and balanced diet. It is an important and strategic nutrient resource since it has low cholesterol and fat ratio compared to red meat and it has healthful properties like easy digestion and being a good source of protein in terms of nutritional value as well as having low cost compared to other protein sources [4]. However, too many animals in poultry-house during broiler breeding causes animals to be more vulnerable and less resistant and thus more susceptible to sudden state changes. Antibiotics, added into rations as feed additive in order to reduce the risk of diseases and achieve rapid live weight gain, played a crucial role until the last few years [5]. However, long-term use of these antibiotics results in the development of resistant strains against antibiotics, thus use of many antibiotics has been prohibited in the many parts of world, especially in the European Union countries since 2006 [6]. Therefore, researchers are in search of a natural and safe growth stimulant that can be an alternative product to maintain developments provided by the use of antibiotics as a growth factor and to overcome the shortcomings that may arise in the absence of antibiotics [7]. For this purpose, the aromatic plants, etheric oils obtained from these plants and their bioactive secondary metabolites are used in medicine, cosmetics and food fields due to their various properties including antibacterial, antioxidant, antiviral, antifungal and digestive stimulatory effect. They are gaining currency to be used as an animal nutrition as well [5]. In plant tissues, high molecular compounds like carbohydrate, lipid, protein, cellulose, lignin and pectin are quite abundant and these substances, called primary (main) metabolites, are necessary for physiological development of plants due to their primary role in cell metabolism [8]. On the other hand, secondary metabolites are produced from primary metabolites via biosynthesis and generally have role in pollination, complying with environmental conditions, chemical defense against microorganisms, insects and other predators and competition with other plants. They are small molecules, whose quantities are sometimes too low to be measured such as alkaloids, volatile oils, glycosides, steroids, flavonoids, tannins, phenols, coloring agent and resins [9, 10]. The bioactive substances synthesized in medicinal and aromatic plants increase or decrease depending on the part of plant which is used as a drug, the physiological period of plant and the time when plant drug are gathered. This change results in difficulty in finding the effective dosage that makes harder use of plant itself or its essential oils directly as feed additive. Therefore, it is preferred to use active substances in the composition of the essential oil isolated from aromatic plants as feed additives [11]. In the experiment, as proportion of carvacrol found in thyme (*Origanum vulgare*) essential oil, cinnamaldehyde found in cinnamon (*Cinnamomum zeylanicum*) essential oils, capsaicin bioactive substances found in chili paprika (*Capsicum annuum*) can vary in etheric oils due to reasons mentioned above, synthetic 99% carvactol, 98% cinnamaldehyde, 99% capsicum oleoresin (includes 6.6% capsaicin), which are identical to natural secondary metabolites were used. Because carvacrol and cinnamaldehyde are both digestive stimulants and can increase appetite as well as being antifunfgal [11, 12], while capsicum oleoresin is endocrine and immune stimulant [13, 14], and also antioxidant [15], lastly the mixture group can act synergistically with various combination of active substances, they were preferred [16]. On the other hand, in studies conducted with different plant extracts and etheric oils, it was found that sensory (tenderness, taste) and physical (toughness, firmness) properties of broiler meat were not affected in general [17], and some of the chemical properties (decreased MDA, increased PUFA) were improved [18]. Indeed, The purpose of this study was to explore the effects of main active ingredients and homogeneous mixtures of carvacrol found in the essential oil of thyme (*Origanum vulgare*), cinnamaldehyde found in the essential oil of cinnamon (*Cinnamomum zeylanicum*) and capsicum oleoresin found in the extracts of hot red pepper (*Capsicum annuum*) on mixed feed at a rate of 150 mg / kg on the sensory, physical and chemical properties of broilers’ breast meat (including pectoralis major and pectoralis minor) and leg meat (including thigh and drumstick).

## Materials and methods

### Chicks, experimental design and diets

A total of 400 male-female broiler chicks (Ross-308) were used as animal material. Experiments were carried out with 5 groups each containing 80 chicks: 4 treatment groups and 1 control group. Each group was further divided into 5 sub-groups each composed of 16 chicks. Among these five dietary treatment groups formed for the trial, the control group was fed without any feed additives. The second group was fed with 150 mg capsicum oleoresin per kg of feed, the third group with 150 mg carvacrol per kg of feed, the fourth group with 150 mg cinnamaldehyde per kg of feed, and the last group with 150 mg mixtures (CAP+CAR+CIN) per kg of feed. The trial was maintained for 6 weeks. Feeds that were used in the experiments were obtained from a commercial feed factory (EGETAV, Izmir, Turkey). Rations were prepared in accordance with the nutrient requirements provided in NRC [19] (Table 1). Rations for each treatment group were prepared by adding 150 mg / kg each of capsicum oleoresin (CAP), carvacrol (CAR), cinnamaldehyde (CIN) or their mixture (CAP+CAR+CIN). To ensure a homogeneous blend, secondary metabolites were pre-mixed with zeolite and the generated crumble was added to the feed. Commercially marketed synthetic 99% carvacrol, 98% cinnamaldehyde and naturally produced 99% capsicum oleoresin and their combinations at a ratio of 1:1:1 were used. The amount of food substance of the feeds used (except for raw cellulose) was specified according to the Weende [20] method while the raw cellulose was specified according to the Lepper method [21]. The regression equation suggested by the 9610 No TSI was used for calculation of the metabolizable energy content [22].

**Table 1:** Ingredients and chemical composition of experimental diets (as-fed basis)

Feed stuff, %	Broiler chick	Broiler
	(0–21. Days)	(22–42. Days)
Corn	46.96	48.91
Soybean meal	20.88	12.37
Wheat	0.00	5
Full-fat soybean	14.57	15
Corn bran	4.5	3
Sunflower seed meal 34% CP	4	4.5
DDGS	2.5	3
Poultry meal	2.29	4
Marble powder	1.12	0.93
DCP 18%	1.08	0.71
Lysine sulfate 70%	0.51	0.46
Commercial fat	0.5	1.15
Commercial methionine	0.32	0.23
^a^Vitamin premix-001	0.2	0.00
^b^Vitamin premix-002	0.00	0.2
Salt	0.19	0.16
Sodium sulfate	0.12	0.10
^c^Mineral	0.10	0.1
L-threonine	0.06	0.04
Liquid choline 75%	0.06	0.06
Coccidiostat	0.05	0.05
Calculated values, g/kg		
Metabolizable energy, kcal/kg	3025	3150
Lysine	14.57	12.72
Methionine + cystine	10.97	9.99
Available P	5.00	4.5
Composition (analyzed), g/kg		
Dry matter	880.29	880.85
Crude protein	237.65	217.67
Ether extract	56.96	66.06
Crude ash	59.73	52.69
Crude fiber	37.06	36.70
Calcium	10.50	9.00
Total phosphorus	6.40	5.69
Starch	344.30	376.12
Sugar	41.24	35.65

^a^Vitamin premix-001 per kg diet: 11000 IU Vitamin A; 5000 IU Vitamin D_3;_ 0.069 mg 25-OH-D_3;_ 150 mg Vitamin E; 3 mg Vitamin K_3;_ 3 mg Vitamin B_1;_ 8 mg Vitamin B_2;_ 4 mg Vitamin B_6;_ 0.02 mg Vitamin B_12;_ 60 mg Niacin; 15 mg D-Pantothenic; 2 mg Folic acid; 0.2 mg Biotin; 100 mg Vitamin C; 400 mg choline,

^b^Vitamin premix-002 per kg diet: There are 10,000 IU Vitamin A; 5000 IU Vitamin D_3;_ 0.069 mg 25-OH-D_3;_ 50 mg Vitamin E; 3 mg Vitamin K_3;_ 3 mg Vitamin B_1;_ 8 mg Vitamin B_2;_ 4 mg Vitamin B_6;_ 0.02 mg Vitamin B_12;_ 60 mg Niacin; 12 mg D-Pantothenic; 2 mg Folic acid; 0.2 mg Biotin; 100 mg Vitamin C; 400 mg choline,

^c^Mineral per kg diet: 150 g Mn, 120 g Fe, 150 g Zn, 14 g Cu, 0,4 g Co, 3 g Se

### Sensory analysis

For sensory analysis, meats were removed from deep freeze 24 h prior to use, thawed at + 4 °C and grilled. Panelists assessed the odor, tenderness, taste, juiciness, appearance and overall acceptability for grilled meat samples scoring from 1 to 5. The assessors were selected and trained according to the international standards [23]. The sensory evaluation was performed according to a standardized sensory descriptive method [24].

### Physical analysis

#### Determination of color specification

Colors of breast meat and leg meat were determined through measurements made by Minolta colorimeter (Minolta CR-300) in CIE L*, a*, b* categories. Measurements were made on 3 different parts of both types of meat. L* value indicated the lightness of the color, while a* value standed for redness and b* value for yellowness [25].

#### Thawing loss

Meat samples of breast and leg which had been weighed during slaughter were removed from deep freezer (− 20 °C) and allowed to stand at + 4 °C overnight before being thawed. The difference between pre-frost and post-defrost weights was calculated as thawing loss [26].

#### Cooking loss

Weighed breast and leg meats were cooked for 45 min in a water bath set at 70 °C. After a short weighing period, they were weighed again after their moisture was removed. Hence, the cooking losses were calculated [27].

#### pH

pH values of the samples of breast and thigh meats were measured with a pH meter (Hanna, USA) at 15^th^ minute (pH 15) after slaughtering.

#### Water holding capacity

Samples of 0.5 g breast and leg meats were placed in aluminum foils between metal plates and their water-holding capacities were calculated by being left underwater at 130 bar pressure for 1 min [28].

#### Texture analysis

To detect cooking losses, samples of breast and leg meats were prepared in dimensions of 3 cm × 1.5 cm × 1 cm (length x width x thickness) and boiled in water. Later, these samples were tested on a TA-XT Plus Texture Analyzer (Stable Micro Systems, Godalming, England), in accordance with the procedures described by Malovrh et al. [29]. In the analysis, a Warner-Bratzler knife, load cell of 50 kg and cylindrical probe of 35 mm diameter were used to measure the toughness and firmness of the meat. Pre-test speed was fixed at 2 mm/s, test speed at 2 mm/s and post-test speed at 10 m/s.

### Chemical analysis

#### Lipid oxidation

Level of malondialdehyde (MDA) as a measure of lipid oxidation in breast and leg meats were determined in accordance with the thiobarbituric acid (TBA) method reported by Witte et al. [30]. Spectrophotometer values of MDA were adjusted by a correction factor (7.8) to calculate milligrams per kilogram of meat [31].

#### Meat Composition

Composition of fatty acids in oil extractions taken from breast and leg meats were determined by an anonymous method [32]. To determine the fatty acid composition, crude fat of the breast and leg meats were extracted using a chloroform:methanol (2:1, vol/vol) mixture according to the method described by Folch et al. [33]. Fatty acids were measured using an HP-6890 gas chromatograph (Hewlett Packard, Palo Alto, CA). The determination of nutrient composition, dry matter, crude ash, crude fat analysis of breast and leg meats (which had been homogenized by shredding), were carried out in accordance with the Weende analysis method [20]. The crude protein contents of breast and thigh meats were determined employing the method developed by Kjeldahly [34]. The amount of nitrogen found as a result of the analysis was multiplied by the factor of 6.25 and the crude protein contents of the samples were found.

### Statistical analysis

SPSS 21.0 [35] program was used for statistical analysis of the data. One-way analysis of variance (ANOVA) was applied to define the effect of the test on sensory, physical and chemical properties of the meat samples while a Duncan test was carried out to determine the difference between the means (*P* < 0.05).

## Results and discussion

### Sensory analysis

Group’s average panel scores according to the flavor of the breast and leg meat are shown in Table 2. It was found out that there was no statistically significant difference in sensory characteristics between groups of breast meat (*P* > 0.05); and the differences were significant for groups of leg meat (*P* < 0.05). The mixed group had the best score in terms of taste, tenderness and juiciness. When literature was reviewed, it was seen that various aromatic compounds had different effects on chicken meat in terms of sensory properties and these effects changed in line with the dosage [36, 37]. Simsek et al. [6] reported that although blends of essential oil (thyme + anise + carnation) at different doses (100, 200 and 400 ppm) did not make any difference in sensory properties, essential oils could be used for antimicrobial use in broiler feeding, especially in adverse environmental conditions, and may have a positive effect on digestion. According to our experimental results, it is possible to say that addition of CAP+CAR+CIN at a dose of 150 mg/kg had a sensory synergistic effect and it improves taste, tenderness, juiciness and overall acceptability.

**Table 2:** Average panel scores according to the flavor of the breast and leg meat of the groups of broiler chicks fed with feed containing capsicum oleoresin, carvacrol, cinnamaldehyde and their mixtures (out of 5, x ± SEM)

	Treatment Groups					
	Control	CAP	CAR	CIN	CAP+CAR+CIN	P
Breast meat						
Taste	3.50 ± 0.31	3.90 ± 0.31	4.00 ± 0.21	3.70 ± 0.30	4.10 ± 0.28	0.712
Tenderness	4.30 ± 0.26	4.10 ± 0.57	3.90 ± 0.31	4.00 ± 0.21	4.00 ± 0.37	0.873
Juiciness	3.50 ± 0.34	3.70 ± 0.34	3.70 ± 0.34	3.30 ± 0.37	3.60 ± 0.31	0.911
Odor	3.60 ± 0.31	3.50 ± 0.31	4.10 ± 0.23	3.70 ± 0.37	3.90 ± 0.23	0.614
Appearance	4.10 ± 0.35	3.90 ± 0.28	3.70 ± 0.34	3.70 ± 0.30	4.60 ± 0.22	0.202
Overall acceptability	3.90 ± 0.18	3.90 ± 0.23	4.10 ± 0.18	3.80 ± 0.20	4.22 ± 0.28	0.649
Leg meat						
Taste	3.67 ± 0.24^b^	4.30 ± 0.26^b^	4.10 ± 0.28^b^	4.11 ± 0.31^b^	5.00 ± 0.00^a^	0.007
Tenderness	4.75 ± 0.16^ab^	4.40 ± 0.22^bc^	4.10 ± 0.23^c^	4.30 ± 0.21^bc^	5.00 ± 0.00^a^	0.012
Juiciness	4.40 ± 0.17^ab^	4.78 ± 0.15^ab^	4.00 ± 0.27^b^	4.40 ± 0.17^ab^	4.90 ± 0.10^a^	0.006
Odor	3.33 ± 0.33	4.13 ± 0.35	3.67 ± 017	3.60 ± 0.31	4.40 ± 0.16	0.052
Appearance	4.44 ± 0.24	4.33 ± 0.29	3.70 ± 0.26	4.60 ± 0.22	4.50 ± 0.27	0.110
Overall acceptability	3.90 ± 0.23^b^	4.30 ± 0.21^b^	4.30 ± 0.26^b^	4.30 ± 0.21^b^	5.00 ± 0.00^a^	0.026

^a, b, c^The differences between means in the same row with different letters are important, *P* < 0.05

Sensory properties (taste, tenderness, juiciness, odor, appearance and overall acceptability) order 1 = Worst, 5 = Best

### Physical analysis

In Table 3, the physical properties of the groups are shown. It was found out that the differences between the groups were statistically significant in terms of thawing, cooking losses and water holding capacity of breast and leg meat (*P* < 0.05). The lowest level of thawing loss was observed in CAR group for breast meat and in CAR and CIN groups for the leg meat while the lowest level of cooking loss was observed in the mixed group that is CAP+CAR+CIN for both breast and leg meats. The lowest level of water holding capacity was found in the CAP and control groups for breast meat, and in CAP group for leg meat (*P* < 0.05). The difference between the treatment groups and the control group was statistically insignificant in terms of pH levels (*P* > 0.05). When the literature on the subject was reviewed, it was seen that addition of essential oils whose main compounds are CAP, CAR or CIN in different ratios had, in general, no effect on the physical properties [38–40]. Elmali et al. [41] stated that the incompatibility of values observed in the study, such as pH levels, cooking loss, color, etc., could be due to the existence of different feeding programs. According to our results of the physical analysis, it can be concluded that the addition of CAR and CIN improves thawing loss in breast and leg meats, the addition of CAP+CAR+CIN improves the cooking loss, and the addition of CAP improves the water holding capacity.

**Table 3:** Effects of capsicum oleoresin, carvacrol, cinnamaldehyde and their mixtures added to broilers’ mixed feed on thawing loss, cooking loss, water holding capacity and pH values of the breast and leg meat (x ± SEM)

		Treatment Groups					
		Control	CAP	CAR	CIN	CAP + CAR + CIN	P
Breast meat	Thawing loss, %	2.88 ± 0.24^c^	2.26 ± 0.27^ab^	1.72 ± 0.19^a^	2.20 ± 0.16^ab^	2.57 ± 0.12^bc^	0.000
	Cooking loss, %	12.18 ± 0.83^bc^	12.63 ± 1.11^bc^	13.15 ± 0.58^c^	9.85 ± 0.52^ab^	7.12 ± 1.71^a^	0.010
	Water holding capacity, %	50.26 ± 1.31^a^	49.37 ± 1.19^a^	43.29 ± 2.63^b^	43.20 ± 0.90^b^	46.16 ± 1.03^ab^	0.010
	pH (15.min.)	5.76 ± 0.04	5.74 ± 0.05	5.74 ± 0.04	5.72 ± 0.06	5.69 ± 0.04	0.823
Leg meat	Thawing loss, %	1.63 ± 0.19^b^	1.63 ± 0.14^b^	0.95 ± 0.14^a^	1.03 ± 0.16^a^	1.31 ± 0.12^ab^	0.011
	Cooking loss, %	18.34 ± 0.95^b^	16.23 ± 1.84^b^	17.17 ± 1.37^b^	15.79 ± 1.01^b^	10.49 ± 1.39^a^	0.024
	Water holding capacity, %	40.31 ± 0.58^c^	46.24 ± 0.90^a^	43.76 ± 0.76^abc^	42.25 ± 1.60^bc^	44.76 ± 1.95^ab^	0.031
	pH (15.min.)	5.88 ± 0.05	5.74 ± 0.05	5.84 ± 0.06	5.81 ± 0.08	5.78 ± 0.06	0.570

^a, b, c^The differences between means in the same row with different letters are important, *P* < 0.05

The color properties of the groups are given in Table 4. Accordingly, the addition CAP, CAR, CIN and CAP+CAR+CIN to the diet at a dose of 150 mg/kg, did not affect the color (L* -lightness, a* -redness, b* -yellowness) of breast meat, but addition of CAP increased the level of L* value while addition of CIN decreased it (*P* < 0.05). Yetisir et al. [42] mentioned that increasing L* value was desirable in terms of acceptance by consumers. Although in our study the statistically highest L* value was found in the CIN group, this fact was not supported by the findings of sensory analysis carried out to determine consumer acceptance. Nevertheless, the sensory characteristics of the mixed group which had an L* value closest to the CIN group, were found to be more favorable.

**Table 4:** Effects of capsicum oleoresin, carvacrol, cinnamaldehyde and their mixtures added to broilers’ mixed feed on the color properties of the breast and leg meats (x ± SEM)

	Treatment Groups						
	Control		CAP	CAR	CIN	CAP + CAR + CIN	P
Breast meat	L*	51.27 ± 0.80	52.46 ± 1.99	52.23 ± 0.99	52.59 ± 1.80	49.98 ± 1.19	0.676
	a*	5.97 ± 0.86	5.52 ± 0.27	5.10 ± 0.62	5.14 ± 0.69	5.52 ± 0.88	0.904
	b*	8.77 ± 0.29	9.75 ± 0.49	8.58 ± 0.73	9.57 ± 0.51	9.95 ± 0.68	0.351
Leg meat	L*	49.18 ± 0.64^abc^	51.06 ± 1.31^c^	50.47 ± 1.24^bc^	46.59 ± 0.62^a^	47.79 ± 0.61^ab^	0.019
	a*	6.65 ± 0.49	5.97 ± 0.67	6.53 ± 0.47	7.17 ± 1.05	6.32 ± 0.39	0.734
	b*	8.53 ± 0.80	9.00 ± 0.62	9.56 ± 0.47	9.39 ± 0.43	9.39 ± 0.64	0.761

^a, b, c^The differences between means in the same row with different letters are important, *P* < 0.05, L* = Lightness, 0 = Black, 100 = White. a* = Redness, Negative values are green, positive values are red. b* = yellowness, Negative values are blue, positive values are yellow

The results of texture analysis for the groups were provided in Table 5. When the groups were compared by the breast meats in terms of firmness, the highest value was found to be in the group with CIN supplement while the lowest value was found to be in the group with CAR supplement. In terms of toughness, no significant difference was observed between groups (*P* > 0.05) except for the group with CAR supplement. On the other hand, in terms of firmness, the difference between treatment groups was found to be statistically insignificant (*P* > 0.05) while the highest value for toughness was found in the CAP+CAR+CIN group and the lowest level of toughness was observed in the control group. In the previous studies, it was reported that the results obtained from texture analysis of chicken meat were in line with the results of sensory or physical analysis (color, odor, taste, etc.) [43–45]. There is an inverse relationship between the firmness value obtained in textural analysis and the tenderness criterion in sensory analysis. However, the results obtained in the experiments indicated that there was no harmony between the firmness levels and tenderness values determined in the groups. Another relationship is between the value of toughness and the ratio of juiciness. The results obtained from the study show that the highest toughness value (in CAP+CAR+CIN treated group) and the best juiciness ratio (again in CAP+CAR+CIN treated group) seem to correlate positively.

**Table 5:** Effects of capsicum oleoresin, carvacrol, cinnamaldehyde and their mixtures added to broilers’ mixed feed on the texture properties of the breast and leg meat (x ± SEM)

		Treatment Groups					
		Control	CAP	CAR	CIN	CAP + CAR + CIN	P
Breast meat	Firmness (kg)	2.00 ± 0.14^bc^	1.98 ± 0.19^bc^	2.38 ± 0.14^c^	1.55 ± 0.09^a^	1.79 ± 0.15^ab^	0.000
	Toughness (kg)	11.09 ± 0.74^a^	11.09 ± 0.79^a^	14.31 ± 0.86^b^	9.32 ± 0.48^a^	9.46 ± 0.82^a^	0.000
Leg meat	Firmness (kg)	2.29 ± 0.14	1.89 ± 0.30	1.75 ± 0.15	1.50 ± 0.15	1.67 ± 0.08	0.166
	Toughness (kg)	16.56 ± 1.59^c^	15.90 ± 1.48^bc^	14.97 ± 1.04^abc^	12.87 ± 1.51^ab^	11.24 ± 0.75^a^	0.008

^a, b, c^The differences between means in the same row with different letters are important, *P* < 0.05

### Chemical analysis

The nutrient compositions of the groups are shown in Table 6. The results indicate that, the addition of the secondary metabolites to ration was effective on crude fat and crude ash levels in breast meat and dry matter as well as crude fat and crude ash levels in leg meat. The highest crude fat ratio for breast meat was found in CIN group, and for leg meat in the control group; the highest proportion of raw protein for breast meat was found in CAR group and for leg meat in CAP+CAR+CIN group. While there was no difference between the groups in terms of dry matter ratio in the breast meat, this ratio for leg meat was highest in CAR group. Kavouridou et al. [46] reported that 10% coconut, palm or flax seed oil extraction were not effective on the chemical composition of broiler chick and so did Fotea et al. [47] for additions of 0.1, 0.3 and 0.7% thyme oil extraction; and Kirkpinar et al. [37] for the additions of 300 mg thyme, garlic, thyme+garlic. Duarte et al. [48] associated the ineffectiveness of 3.3, 6.6 and 9.9% of corn oil additions on CF of the chicken meat with the level of energy intake; and claimed that it could be due to the fact that the energy value of the feed had not changed.

**Table 6:** Effects of capsicum oleoresin, carvacrol, cinnamaldehyde and their mixtures added to broilers’ mixed feed on the nutrient compositions of the breast and leg meat (x ± SEM)

		Treatment Groups					
		Control	CAP	CAR	CIN	CAP + CAR + CIN	P
Breast meat	Dry matter,%	26.57 ± 0.25	26.28 ± 0.26	26.68 ± 0.17	26.37 ± 0.28	26.89 ± 0.26	0.424
	Crude ash,%	0.95 ± 0.10	1.20 ± 0.04	1.07 ± 0.07	1.15 ± 0.06	1.10 ± 0.07	0.097
	Crude fat,%	0.66 ± 0.09^a^	0.26 ± 0.03^b^	0.35 ± 0.04^b^	0.73 ± 0.06^a^	0.36 ± 0.08^b^	0.000
	Crude protein,%	24.93 ± 0.26^bc^	25.02 ± 0.25^bc^	27.61 ± 1.00^a^	23.95 ± 0.23^c^	25.41 ± 0.32^b^	0.000
Leg meat	Dry matter,%	23.34 ± 0.27^b^	24.26 ± 0.35^b^	25.86 ± 0.61^a^	23.93 ± 0.71^b^	24.23 ± 0.45^b^	0.009
	Crude ash,%	1.06 ± 0.06	0.93 ± 0.04	1.03 ± 0.04	1.04 ± 0.03	1.07 ± 0.05	0.140
	Crude fat,%	0.80 ± 0.06^a^	0.48 ± 0.08^bc^	0.65 ± 0.08^ab^	0.55 ± 0.08^b^	0.28 ± 0.05^c^	0.000
	Crude protein,%	21.10 ± 0.25^b^	20.41 ± 0.28^b^	21.23 ± 0.44^b^	21.41 ± 0.35^b^	23.52 ± 0.76^a^	0.001

^a, b, c^The differences between means in the same row with different letters are important, *P* < 0.05

In Table 7, lipid oxidation values for the groups on day 1, day 4 and day 8 are presented. It was observed that the effects of the addition of CAP, CAR, CIN or CAP+CAR+CIN at a dose of 150 mg / kg on the lipid oxidation in breast and leg meat on day 1, day 4 and day 8 were not statistically significant compared to control group (*P* > 0.05). Symeon et al. [40] reported that the addition of 0.5–1.0 mL/kg of cinnamon oil had no effect on MDA level in the *pectoralis major* muscles taken from chicks on days 1, 3, 5 and 9 as well as in the 4th, 5th and 6th months. Luna et al. [49] stated that addition of 150 mg/kg thymol and 150 mg/kg carvacrol improves MDA values in breast and leg meat. They claimed that this effect for breast meat was more prominent on day 5, partial on day 10, and for leg meat it is partial on day 5 and more prominent on day 10. Ciftci et al. [18] stated that addition of 1000 ppm cinnamon oil improved MDA value relative to control group, which could be due to antioxidant property of cinnamon oil. Faix et al. [50] associated the fact that the addition of 250 ppm - 1000 ppm cinnamon oil reduced the MDA level in the blood serum to the reduction of lipid oxidation through increase of hepatic antioxidant enzyme activities (glutathioneeproxidase, superoxidedismutase, catalase, gamma-glutamyltransferase, etc.). Regarding the secondary metabolites that make up the working material, Kim et al. [51] and Lillehoj et al. [52] reported that they were effective on antibacterial activity and on lipid metabolism. According to the results of our study, CAP, CAR and CIN generally affected lipid oxidation in samples taken from breast and leg meats, but this effect did not appear statistically significant. However, it is the combination of CAP+CAR+CIN that most positively affected lipid oxidation in both breast and leg meat although this effect remained at just numerical level.

**Table 7:** Effects of capsicum oleoresin, carvacrol, cinnamaldehyde and their mixtures added to broilers’ mixed feed on the lipid oxidation of the breast and leg meat (x ± SEM)

		Treatment Groups					
		Control	CAP	CAR	CIN	CAP + CAR + CIN	P*
Breast meat	1.day	0.45 ± 0.07	0.43 ± 0.05	0.39 ± 0.04	0.36 ± 0.03	0.29 ± 0.03	0.193
	4.day	0.47 ± 0.06	0.44 ± 0.07	0.45 ± 0.06	0.70 ± 0.10	0.33 ± 0.08	0.700
	8.day	0.85 ± 0.26	0.91 ± 0.47	0.77 ± 0.40	0.76 ± 0.17	0.73 ± 0.18	0.165
Leg meat	1.day	0.57 ± 0.13	0.63 ± 0.10	0.49 ± 0.05	0.60 ± 0.10	0.39 ± 0.03	0.553
	4.day	0.80 ± 0.13	0.74 ± 0.10	0.64 ± 0.06	0.70 ± 0.10	0.43 ± 0.01	0.157
	8.day	0.99 ± 0.26	0.87 ± 0.51	0.74 ± 0.46	0.74 ± 0.08	0.68 ± 0.18	0.930

*P < 0.05

In Table 8, the fat compositions of the breast meat in different groups are illustrated. Expectedly, the difference between the groups was found to be statistically significant for linoleic, eicosanoid, linolenic and lignoseric acids (*P* < 0.05). When the groups were evaluated for total fatty acid contents of breast meat and Σn-3, Σn-6 Σn-9 fatty acids, it was found out that the difference between total monounsaturated fatty acids (MUFA) and Σn-3 fatty acids was statistically significant (*P* < 0.05) while the difference between total saturated (SFA), unsaturated (UFA), polyunsaturated (PUFA), Σn-6 and Σn-9 fatty acids was not statistically significant (*P* > 0.05). Highest ratio for ΣMUFA was observed in CAR group while values for eicosanoid, eicosapentaenoic acid (EPA), lignoseric and Σn-3 fatty acids were found to be highest in CAP+CAR+CIN group. Bolukbasi et al. [53] reported that adding 100 and 200 mg/kg of thyme oil to the feed decreased the ratio of ΣSFA while the same addition increased ΣMUFA and ΣPUFA ratios in breast meat. Ciftci et al. [18] reported that the addition of 500 ppm and 1000 ppm cinnamon oil to the broilers’ ration reduced the ratio of ΣSFA but significantly increased the ratio of Σn-3 and Σn-6 fatty acids, without any affect on ΣMUFA in breast meat. Shin et al. [54] reported that addition of conjugated linoleic acid, flaxseed oil or fish oil at a rate of 2% in ration of broiler chickens decreased PUFA while increasing Σn-3 and Σn-6 levels. Sadeghi et al. [55] reported that addition of fish oil, instead of corn, to the ration at a rate of 5% did not affect SFA, MUFA, PUFA, but decreased Σn-6 and increased EPA and Σn-3 levels in breast meat.

**Table 8:** Effects of capsicum oleoresin, carvacrol, cinnamaldehyde and their mixtures added to broilers’ mixed feed on the composition of fatty acids in the breast meat (x ± SEM)

	Treatment Groups					
Fatty acids of breast meat^1^	Control	CAP	CAR	CIN	CAP+CAR+CIN	P
C_12:0_ Lauric	0.05 ± 0.03	0.22 ± 0.18	0.15 ± 0.11	0.03 ± 0.03	0.31 ± 0.15	0.291
C_14:0_ Myristic	0.28 ± 0.08	0.72 ± 0.36	0.64 ± 0.18	0.21 ± 0.10	0.39 ± 0.04	0.121
C_16:0_ Palmitic	18.65 ± 0.57	13.20 ± 6.22	18.36 ± 0.17	17.69 ± 0.71	17.50 ± 0.57	0.312
C_16:1_Palmitoleic	1.82 ± 0.27	1.61 ± 0.26	3.27 ± 0.93	1.89 ± 0.38	1.85 ± 0.27	0.254
C_17:0_ Margaric	0.53 ± 0.04	0.27 ± 0.14	0.48 ± 0.13	0.53 ± 0.46	0.42 ± 0.12	0.973
C_17:1_ Heptadecenoic	0.00 ± 0.00	0.10 ± 0.1	0.05 ± 0.05	0.91 ± 0.91	0.40 ± 0.32	0.662
C_18:0_ Stearic	6.78 ± 0.29	7.24 ± 0.60	6.32 ± 0.22	5.65 ± 0.63	5.86 ± 0.45	0.191
C_18:1n-9_ Oleic	30.09 ± 0.00	28.22 ± 0.26	30.27 ± 1.21	29.07 ± 0.98	28.78 ± 0.67	0.582
C_18:3n-6_ Linoleic	37.26 ± 1.17^a^	37.38 ± 1.05^a^	34.86 ± 0.76^ab^	37.93 ± 0.90^a^	33.07 ± 1.68^b^	0.030
C_18:3n-6_ Translinoleic	0.00 ± 0.00	0.15 ± 0.15	0.07 ± 0.03	0.09 ± 0.09	0.01 ± 0.01	0.560
C_20:0_ Arachidonic	1.25 ± 0.66	0.04 ± 0.04	0.55 ± 0.39	1.19 ± 0.53	0.34 ± 0.30	0.402
C_20:1_ Eicosanoid	0.06 ± 0.06^b^	0.12 ± 0.1^b^	0.15 ± 0.1^b^	0.01 ± 0.00^b^	0.87 ± 0.34^a^	0.006
C_20:1n-9_ Linolenic	0.90 ± 0.55	2.25 ± 0.25	1.80 ± 0.36	0.95 ± 0.45	1.61 ± 0.42	0.263
C_20:1n-9_ Translinolenic	0.31 ± 0.10	0.26 ± 0.15	0.14 ± 0.07	0.14 ± 0.07	0.38 ± 0.26	0.641
C_20:5n-3_ EPA	0.00 ± 0.00^b^	0.00 ± 0.00^b^	0.01 ± 0.01^b^	0.03 ± 0.01^b^	0.78 ± 0.41^a^	0.030
C_20:6n-3_ DHA	0.58 ± 0.22	0.15 ± 0.1	0.05 ± 0.05	0.08 ± 0.05	1.07 ± 0.61	0.050
C_22:0_ Behenic	1.58 ± 0.17	1.73 ± 0.13	0.14 ± 0.27	2.67 ± 0.99	2.02 ± 0.13	0.352
C_22:5n-3_ DPA	0.00 ± 0.00	0.00 ± 0.00	0.12 ± 0.07	0.00 ± 0.00	0.09 ± 0.09	0.701
C_24:0_ Lignoseric	0.48 ± 0.32^b^	0.41 ± 0.21^b^	0.42 ± 0.22^b^	0.12 ± 0.08^b^	1.36 ± 0.17^a^	0.005
C_24:1n-9_ Nervonic	0.47 ± 0.39	0.11 ± 0.07	0.22 ± 0.18	0.23 ± 0.36	0.93 ± 0.36	0.330
∑SFA	29.60 ± 1.21	30.82 ± 0.79	28.07 ± 0.73	28.10 ± 0.76	28.13 ± 0.70	0.331
∑UFA	69.73 ± 1.15	70.06 ± 1.61	70.94 ± 0.56	71.10 ± 1.01	69.21 ± 1.84	0.758
∑MUFA	31.14 ± 0.45^bc^	30.15 ± 0.48^c^	33.97 ± 0.69^a^	32.11 ± 0.54^abc^	32.08 ± 0.60^ab^	0.005
∑PUFA	37.29 ± 1.78	39.90 ± 1.17	36.97 ± 0.73	38.99 ± 1.02	36.41 ± 1.94	0.455
∑n-3	1.96 ± 0.37^b^	2.53 ± 0.13^ab^	2.53 ± 0.23^ab^	1.90 ± 0.35^b^	3.67 ± 0.59^a^	0.039
∑n-6	37.45 ± 0.27	37.38 ± 1.05	35.57 ± 0.35	37.93 ± 0.90	35.14 ± 1.79	0.257
∑n-9	30.56 ± 1.15	28.33 ± 0.27	30.50 ± 1.05	29.31 ± 0.95	29.68 ± 0.64	0.581

^a, b, c^The differences between means in the same row with different letters are important, *P* < 0.05

^1^Breast meat in % fatty acid

In Table 9, the fat compositions of the leg meat in different groups are presented. The differences found for linoleic, EPA and behenic fatty acids are statistically important (*P* < 0.05). When the groups were evaluated according to their total fatty acid content and Σn-3, Σn-6 Σn-9 fatty acid levels in leg meat, it was determined that the differences between groups in terms of MUFA, PUFA and Σn-6 fatty acid levels were statistically significant (*P* < 0.05) while differences in terms of SFA, UFA, Σn-3 and Σn-9 fatty acids were not statistically significant (*P* > 0.05). The highest ΣMUFA ratio was found in CAP+CAR+CIN group while the highest ΣPUFA ratio was observed both in CAP and control groups. Σn-6 fatty acids values were at highest levels in CAP group. Bolukbasi et al. [53] reported that the addition of 100 mg/kg and 200 mg/kg of thyme oil to the feed lowered the ratio of ΣSFA in comparison to the control group, while the ratio of ΣPUFA increased in line with the dosage. Shin et al. [54] reported that addition of conjugated linoleic acid, linseed oil or fish oil to the ration at a rate of 2% decreased MUFA level in leg meat while increasing PUFA and Σn-3 values in comparison with the control group. Sadeghi et al. [55] reported that addition of fish oil instead of corn at a rate of 5% to the ration at different weeks (2, 3, 4, 5 and 6) increased SFA and Σn-3 in leg meat in 4th week in comparison to control group, reduced Σn-6 ratio but did not change MUFA and PUFA values. ΣMUFA and ΣPUFA are required to be taken with food as ΣMUFA is an HDL (High Density Lipoprotein) enhancer with beneficial effects on health, and ΣPUFA has positive effects on some body functions and blood. The results obtained through our study indicated that addition of CAR increased ΣMUFA ratio in the breast meat; addition of CAR, CIN, CAP+CAR+CIN decreased ΣPUFA ratio in the breast meat; and addition of CAP did not change the values in the breast meat and it did not have any effect at all on the leg meat. On the other hand, omega fatty acids, which are not synthesized in the body, are required to be consumed with nutrients because of their positive effects on brain development, strengthening the immune system and prevention of heart diseases [56, 57]. The results of our study revealed that omega fatty acids in breast meat and leg meat in treatment groups were positively affected. Accordingly, it was determined that the addition of CAP, CAR, CIN or CAP+CAR+CIN to the ration at a rate of 150 mg/kg changed the profile of fatty acids positively in broiler chickens, with different effect levels on their accumulation in breast meat and leg meat.

**Table 9:** Effects of capsicum oleoresin, carvacrol, cinnamaldehyde and their mixtures added to broilers’ mixed feed on the composition of fatty acids in leg meat (x ± SEM)

	Treatment Groups					
Fatty acids of leg meat^1^	Control	CAP	CAR	CIN	CAP+CAR+CIN	P
C_12:0_ Lauric	0.18 ± 0.15	0.08 ± 0.03	0.22 ± 0.11	0.34 ± 0.27	0.07 ± 0.03	0.688
C_14:0_ Myristic	0.35 ± 0.01	0.39 ± 0.05	0.47 ± 0.05	0.60 ± 0.21	0.53 ± 0.14	0.645
C_16:0_ Palmitic	15.10 ± 4.34	12.20 ± 4.61	18.77 ± 0.94	16.67 ± 0.29	18.20 ± 0.38	0.312
C_16:1_ Palmitoleic	1.29 ± 0.65	1.93 ± 0.02	2.10 ± 0.56	2.29 ± 0.47	2.87 ± 0.03	0.317
C_17:0_ Margaric	0.19 ± 0.1	0.35 ± 0.13	0.39 ± 0.07	0.70 ± 0.39	0.72 ± 0.52	0.749
C_17:1_ Heptadecenoic	0.04 ± 0.04	0.07 ± 0.04	0.05 ± 0.02	0.02 ± 0.02	0.06 ± 0.02	0.730
C_18:0_ Stearic	4.36 ± 2.18	5.87 ± 0.33	4.32 ± 1.12	5.55 ± 4.65	5.50 ± 0.21	0.817
C_18:1n-9_ Oleic	32.31 ± 2.58	28.40 ± 0.07	31.95 ± 1.82	29.83 ± 0.84	32.23 ± 0.30	0.515
C_18:3n-6_ Linoleic	37.45 ± 0.27^bc^	40.22 ± 1.02^a^	36.21 ± 0.72^bc^	37.97 ± 1.28^ab^	34.98 ± 0.58^c^	0.014
C_18:3n-6_ Translinoleic	0.12 ± 0.12	0.00 ± 0.00	0.01 ± 0.01	0.06 ± 0.06	0.01 ± 0.01	0.523
C_20:0_ Arachidonic	0.72 ± 0.72	1.10 ± 1.07	1.36 ± 0.54	1.48 ± 0.67	1.12 ± 0.54	0.939
C_20:1_ Eicosanoid	0.31 ± 0.27	0.02 ± 0.02	0.15 ± 0.04	0.27 ± 0.11	0.21 ± 0.05	0.617
C_20:1n-9_ Linolenic	1.45 ± 0.73	1.23 ± 1.23	0.94 ± 0.58	0.77 ± 0.77	1.10 ± 0.63	0.973
C_20:1n-9_ Translinolenic	0.05 ± 0.05	0.22 ± 0.00	0.05 ± 0.03	0.19 ± 0.03	0.17 ± 0.09	0.143
C_20:5n-3_ EPA	0.01 ± 0.01^b^	0.00 ± 0.00^b^	0.32 ± 0.10^a^	0.43 ± 0.10^a^	0.02 ± 0.01^b^	0.001
C_20:6n-3_ DHA	0.19 ± 0.10	0.22 ± 0.02	0.25 ± 0.10	0.50 ± 0.29	0.23 ± 0.12	0.654
C_22:0_ Behenic	1.74 ± 0.00^a^	1.65 ± 0.06^a^	1.80 ± 0.06^a^	1.14 ± 0.06^b^	1.20 ± 0.06^b^	0.001
C_22:5n-3_ DPA	0.00 ± 0.00	0.00 ± 0.00	0.29 ± 0.19	0.42 ± 0.26	0.27 ± 0.16	0.527
C_24:0_ Lignoseric	0.41 ± 0.30	0.42 ± 0.09	0.41 ± 0.17	0.26 ± 0.13	0.42 ± 0.05	0.964
C_24:1n-9_ Nervonic	0.24 ± 0.24	0.07 ± 0.07	0.28 ± 0.15	0.39 ± 0.25	0.12 ± 0.05	0.748
∑SFA	26.17 ± 1.19	26.22 ± 0.13	27.38 ± 0.38	26.38 ± 0.40	27.76 ± 0.80	0.240
∑UFA	73.29 ± 0.86	72.14 ± 0.15	72.46 ± 0.39	72.75 ± 0.72	72.13 ± 0.45	0.578
∑MUFA	32.53 ± 0.49^bc^	30.48 ± 0.02^b^	33.54 ± 0.80^ab^	32.80 ± 1.10^bc^	35.54 ± 0.71^a^	0.004
∑PUFA	39.10 ± 0.87^a^	41.65 ± 0.13^a^	38.62 ± 0.71^b^	38.39 ± 1.41^b^	36.59 ± 0.69^b^	0.008
∑n-3	1.65 ± 0.69	1.45 ± 0.72	1.62 ± 0.90	1.98 ± 0.88	1.61 ± 0.88	0.996
∑n-6	37.45 ± 0.27^b^	40.22 ± 0.59^a^	36.21 ± 0.72^bc^	37.97 ± 1.28^ab^	34.98 ± 0.58^c^	0.001
∑n-9	32.55 ± 2.42	28.46 ± 0.00	32.35 ± 1.70	30.22 ± 0.61	32.41 ± 0.30	0.275

^a, b, c^The differences between means in the same row with different letters are important, *P* < 0.05

^1^Leg meat in % fatty acid

## Conclusion

In conclusion, after the prohibition of antibiotics in poultry nutrition, research on the potential use of natural supplements has been accelerated in order to improve the performance and meat quality. In this study, the effects of dietary supplementation of the main active ingredients of different essential oils and extracts on sensory, physical and chemical properties of breast meat and leg meat were investigated. Consequently, it was determined that addition of CAP, CAR, CIN or CAP+CAR+CIN at a rate of 150 mg/kg affected the sensory properties of taste, tenderness, juiciness and overall acceptability; the physical properties of L* value and toughness; and chemical properties of DM, CF, CP, linoleic, EPA, behenic, MUFA, PUFA, Σn-6 of the breast meat. Supplementation of these components also affected the physical properties of toughness and firmness as well as the chemical properties of CF, CP, linoleic, eicosanoid, EPA, lignoseric, MUFA and Σn-3 of the leg meat. Furthermore, while the treatments had a positive effect on both breast meat and leg meat with respect to thawing loss, cooking loss and water holding capacity; no effects were observed over pH value and lipid oxidation on day 1, day 4 and day 8. The results of the study indicated that addition of CAP, CAR, CIN or CAP+CAR+CIN to the ration improves the quality of the meat. However, the overall results suggest using mixtures of active ingredients to achieve the best effect in practice.

## Abbreviations

∑n-3: Total Omega-3 Fatty Acids
∑n-6: Total Omega-6 Fatty Acids
°C: Centigrade
a*: Redness
b*: Yellowness
CAP: Capsicum oleoresin
CAR: Carvacrol
CF: Crude Fat
CIN: Cinnamaldehyde
CP: Crude Protein
D: Dry Matter
EPA: Eicosapentaenoic Acid
HDL: High Density Lipoprotein
kg: Kilogram
L*: Lightness
MDA: Malondialdehyde
mg: Milligram
MUFA: Monounsaturated Fatty Acids
PUFA: Polyunsaturated Fatty Acids
SFA: Saturated Fatty Acids
TBA: Thiobarbituric Acid
UFA: Unsaturated Fatty Acids
Σn-9: Total Omega-9 Fatty Acids
